# Dietary *p*-Coumaric Acid Modulates Non-Core Gut Microbiota and Sucrose Solution Consumption in *Apis cerana*

**DOI:** 10.3390/insects17040371

**Published:** 2026-04-01

**Authors:** Haodong Wu, Conghui Ji, Kun Dong, Ruisheng Wang, Lijiao Gao, Wenhua Luo, Jialin Liu

**Affiliations:** 1 Institute of Economic Animal, Chongqing Academy of Animal Sciences, Chongqing 402460, China; wuhaodong177@163.com (H.W.); j1621042024@163.com (C.J.); 13436082757@163.com (R.W.); gaolijiao002004@163.com (L.G.); luowh1966@126.com (W.L.); 2 Yunnan Provincial Engineering and Research Center for Sustainable Utilization of Honey Bee Resources, Eastern Bee Research Institute, College of Animal Science and Technology, Yunnan Agricultural University, Kunming 650201, China; dongkun19722004@aliyun.com

**Keywords:** *p*-coumaric acid, honeybee, gut microbial modulation, absolute 16S rRNA sequencing, sucrose solution consumption

## Abstract

As the predominant native pollinator across Asia, *Apis cerana* is essential for the maintenance of biodiversity and agricultural productivity. The gut microbiota of honeybees plays a central role in host nutrition, detoxification, and immune function. *p*-Coumaric acid, a widespread phenolic acid enriched in pollen and nectar, has been reported to promote honeybee health by prolonging lifespan and increasing the expression of detoxification-related genes, hence improving tolerance to pesticides. Its influence on gut microbial communities, however, remains insufficiently characterized in *A. cerana*. This study evaluated the effects of dietary *p*-coumaric acid on survival, sucrose solution consumption, and gut microbiome composition in *A. cerana* workers using absolute quantification sequencing. Bees were provided sucrose solutions containing *p*-coumaric acid at concentrations of 41.0, 82.0, and 164.0 mg/L for durations of 5 and 10 days. The results indicated no effect on survival but revealed time-dependent changes in sucrose solution consumption. *p*-Coumaric acid exposure altered the abundance of non-core bacterial taxa, including *Bombella* and *Apilactobacillus*, whereas the core gut microbiota (*Lactobacillus*, *Gilliamella*, *Snodgrassella*, *Apibacter*, and *Bifidobacterium*) remained stable. These results suggest that *p*-coumaric acid modulates sucrose solution consumption and selectively influences non-core gut bacteria without disrupting survival or core microbiota stability, underscoring its role in regulating host–microbe interactions in honeybees.

## Simple Summary

Dietary exposure of honey bees to *p*-coumaric acid, a phenolic compound naturally present in pollen, nectar and propolis, is routinely encountered by honeybees during foraging activities. It has been associated with increased detoxification capacity and overall health. The extent to which *p*-coumaric acid affects honeybee health through alterations in the gut microbiota remains unclear. This study demonstrated that dietary supplementation with *p*-coumaric acid for 5 and 10 days did not reduce the survival of *Apis cerana* workers but induced temporal changes in sucrose solution consumption. While the core gut bacterial community remained essentially unchanged, several non-core taxa, including *Bombella* and *Apilactobacillus*, showed specific responses. Our findings provide new insights into how naturally occurring phytochemicals shape the gut microbiome and support honeybee health.

## 1. Introduction

Bee pollinators play an indispensable role in sustaining agricultural productivity and ecosystem stability [1,2]. They contribute to the reproduction of approximately 75% of major global food crops [3], supporting improvements in yield, quality, and uniformity of fruits, vegetables, oilseeds, and nuts [4,5]. Beyond agriculture, bees are important in sustaining plant diversity in natural ecosystems, as animal pollinators are required for an estimated 78% of temperate and 94% of tropical plant species, respectively [6,7]. Despite their ecological and economic importance, pollinator populations, particularly bees, are experiencing significant global declines under the combined pressures of habitat loss, intensive pesticide application, climate change, and the increasing prevalence of pathogens and parasites [8,9,10]. These reductions pose significant risks to food security and ecological resilience by destabilizing plant–pollinator interactions and limiting genetic diversity within plant communities [11,12]. The estimated annual economic value of pollination services, ranging from US$ 235 to 577 billion [13], highlights the urgency of addressing these challenges. There is a pressing need to strengthen research efforts to protect bee populations, improve pollinator health, and incorporate pollinator-supportive approaches into agricultural management and environmental conservation [14]. Such measures are essential for maintaining ecosystem services and supporting the long-term sustainability of global food systems.

A key determinant of honeybee health is the gut microbiome, a specialized bacterial community that performs essential functions in host nutrition, detoxification, immune regulation, and defense against pathogens [15]. The honeybee gut contains a relatively simple yet highly stable microbial consortium. *Apis cerana* and *Apis mellifera* share a core group of four bacterial genera, *Snodgrassella*, *Gilliamella*, *Lactobacillus*, and *Bifidobacterium* [16]; however, their overall community compositions differ. One significant distinction is *Apibacter*, which is frequently detected in *A. cerana* but rarely observed in *A. mellifera* [17,18]. Gut microbiota is transmitted socially to newly emerged workers through interactions with nestmates and contact with hive surfaces, after which they colonize defined niches within the hindgut at high abundances [19]. This microbial community supports honeybee health by degrading complex pollen-derived polysaccharides, mediating the detoxification of xenobiotics, and activating host immune responses [20,21,22,23]. Moreover, the gut microbiota also influences development and behavior through regulation of endocrine signaling and neuroactive pathways [24,25,26]. Both the composition and functional capacity of this community are strongly shaped by dietary quality. Nutritional deficiency is associated with microbial imbalance, reduced abundance of core symbionts, and increased susceptibility to pathogens such as *Nosema ceranae* [22,27]. The gut microbiota, therefore, represents a central interface linking nutrition with overall honeybee health [28,29].

Phytochemicals are non-nutritive, bioactive secondary metabolites of plant origin that are routinely encountered by honeybees through floral resources such as nectar, pollen, and propolis, where they exert significant effects on bee physiology [30]. Among these compounds, phenolic acids are particularly prevalent and represent integral components of both pollen biology and the apian diet [31,32,33]. *p*-Coumaric acid is a phenolic acid that functions as a monomeric precursor of sporopollenin in the pollen exine [31,32]. It is also widely present in nectar [33,34], rendering it a prominent and biologically relevant dietary constituent for honeybees. Dietary intake of *p*-coumaric acid has been shown to elevate the expression of detoxification-related genes in *A. mellifera*, especially cytochrome P450 monooxygenases of the CYP9Q subfamily, increasing metabolic capacity for the biotransformation of synthetic pesticides [35]. Previous studies have reported that *p*-coumaric acid reduces *N. ceranae* spore loads in *A. mellifera* [36,37], potentially via activation of immune signaling pathways and upregulation of antimicrobial peptide synthesis [38]. *p*-Coumaric acid has also been associated with increased longevity through attenuation of oxidative stress and regulation of hormone signaling pathways linked to aging [30,35,39,40]. Recent findings further suggest that *p*-coumaric acid shapes the gut microbiota by promoting the abundance of beneficial core bacterial taxa, including *Snodgrassella* and *Lactobacillus*, which are involved in metabolic homeostasis and pathogen resistance in *A. mellifera* [30]. These combined effects underscore the importance of dietary phytochemicals in maintaining honeybee health and reflect a co-evolved mutualistic relationship between flowering plants and their pollinators [41,42,43].

Despite the well-documented physiological benefits of *p*-coumaric acid, its specific effects on the gut microbiome of the native Asian honeybee, *A. cerana*, remain insufficiently defined. As key pollinators supporting biodiversity and agricultural productivity across Asia, *A. cerana* populations are experiencing declines in multiple regions due to interacting stressors, including habitat degradation, pesticide exposure, and pathogen burden [9,44]. Elucidating how *A. cerana* responds to dietary phytochemicals is therefore essential for the development of effective strategies to protect this native species and maintain its pollination services. The distinct gut microbial composition of *A. cerana* suggests that its response to *p*-coumaric acid may differ from that of *A. mellifera*, although previous evidence indicates that *p*-coumaric acid promotes gut microbial growth in *A. mellifera*. This emphasizes the need for a species-specific evaluation of *p*-coumaric acid–microbiome interactions in *A. cerana*. Moreover, unlike earlier studies that reported only relative bacterial abundance [30], the present employed absolute quantification sequencing, enabling more precise characterization of genuine changes in bacterial load and community composition.

In light of the significant differences in gut microbial composition between *A. cerana* and *A. mellifera*, it was hypothesized that dietary *p*-coumaric acid exerts species-specific effects on the gut microbiota of *A. cerana*. To address this, the study was designed with the following objectives: (i) to determine the effect of *p*-coumaric acid supplementation on the survival of *A. cerana* workers; (ii) to evaluate whether dietary *p*-coumaric acid influences sucrose solution consumption; and (iii) to characterize the effects of dietary *p*-coumaric acid on gut microbial community structure and bacterial abundance using absolute quantification sequencing. The findings provide insight into the microbiome-level responses of *A. cerana* to dietary phytochemicals and establish a basis for developing conservation strategies tailored to this ecologically and agriculturally important pollinator.

## 2. Materials and Methods

### 2.1. Experimental Honeybees and Treatments

Worker bees (*A. cerana*) used in this study were sourced from three healthy colonies, each headed by a naturally mated sister queen and maintained on five-frame hives. Newly emerged workers were collected, age-synchronized, marked, and colonized in cages according to the previously described protocol [45]. After an acclimation period and 2 h of starvation, bees were assigned to dietary treatments. The experiment included four dietary treatment groups: a control group (CK) receiving a sterile 50% (*w/w*) sucrose solution containing 0.25% dimethyl sulfoxide (DMSO, Macklin Co., Ltd., Shanghai, China), and three *p*-coumaric acid groups receiving the same sucrose solution supplemented with *p*-coumaric acid (Sigma-Aldrich Co., Saint Louis, MO, USA) at final concentrations of 41.0, 82.0, and 164.0 mg/L, each also containing 0.25% DMSO. The selected concentration range corresponds to naturally occurring levels of *p*-coumaric acid in honeybee diets [33], and previous studies demonstrated that 82.0 mg/L can extend the lifespan of both *A. cerana* and *A. mellifera* [35,46]. Bees in all groups were maintained under their respective treatments for 5 or 10 consecutive days in a controlled environment set at 30 °C and 60% relative humidity. Each treatment consisted of five biological replicate cages, with 40 bees per cage, totaling 1600 bees per independent experimental cohort. Two fully independent cohorts were used in parallel: one for survival and sucrose consumption assays, and the other for gut microbiota analysis. The experimental design encompassed 3200 individual bees across all treatment groups and cohorts.

### 2.2. Effect of p-Coumaric Acid on Worker Survival and Sucrose Solution Consumption

Following a 5- or 10-day dietary treatment, all groups were provided with sterile sucrose solution, and survival was monitored daily until all individuals had died. Daily sucrose consumption was measured over 30 days, encompassing both the treatment and post-treatment phases. Daily maintenance included replenishing feeding solutions, weighing feeders, recording mortality, and removing dead individuals. Dietary intake was determined gravimetrically by measuring feeder mass before and after exposure, with evaporative losses corrected using parallel control cages containing identical solutions. Corrected mass loss was normalized to the number of surviving bees to calculate mean daily consumption per bee.

### 2.3. Gut Microbiota DNA Extraction, 16S rRNA Sequencing, and Bioinformatics Analysis

Whole gut samples were collected from worker bees for microbiota analysis. For each group, 25 individuals were dissected, and the guts were pooled to generate five biological replicates, with each replicate consisting of five guts. Total microbial genomic DNA was extracted from these pooled gut samples using the E.Z.N.A.^®^ Soil DNA Kit (Omega Bio-tek, Norcross, GA, USA). For absolute quantification analysis, 12 synthetic spike-in sequences, ranging from 10^3^ to 10^6^ copies and consisting of artificial variable regions with minimal similarity to reference databases, were added to each DNA sample. The V3-V4 hypervariable region of the bacterial 16S rRNA gene was amplified using primers 338F/806R on a T100 Thermal Cycler (BIO-RAD, Hercules, CA, USA). PCR amplicons were purified with the PCR Clean-Up Kit (YuHua, Shanghai, China), quantified using a Qubit 4.0 fluorometer, pooled in equimolar concentrations, and sequenced in paired-end mode on an Illumina NextSeq2000 platform (Majorbio Bio-Pharm, Shanghai, China). Raw sequencing data are available in the NCBI SRA under accession PRJNA1399622. Processing of reads included demultiplexing, quality filtering with fastp v0.19.6 [47], and merging with FLASH v1.2.11 [48]. Denoising and amplicon sequence variant (ASV) inference were performed in QIIME2 (v2020.2) using DADA2 [49] with default settings. ASVs derived from spike-in sequences were removed before generating sample-specific standard curves (read counts versus spike-in DNA copies; Table S1) for absolute abundance estimation. Taxonomic assignment was conducted by aligning sequences to the SILVA v138 database using the QIIME2 naive Bayes classifier, followed by correction for rRNA operon copy number based on rrnDB [50,51].

### 2.4. Statistical Analysis

All statistical analyses were performed using SPSS 30.0 (IBM, Armonk, NY, USA). Survival differences were assessed using Kaplan–Meier analysis, with treatment effects evaluated via the log-rank test and Bonferroni correction. Total sucrose solution consumption was analyzed using two-way repeated-measures ANOVA after confirming that the assumptions of normality, homogeneity, and sphericity were satisfied. Mean sucrose consumption per treatment was further compared using one-way ANOVA followed by Tukey HSD post hoc tests. Microbial diversity was assessed by calculating alpha diversity indices (Shannon, Simpson, Chao1) from ASV data using Mothur v1.30.1. Beta diversity was evaluated through Bray–Curtis dissimilarity and visualized via Principal coordinate analysis (PCoA) using the Vegan v2.5-3 package in R 3.3.1, with permutational multivariate ANOVA applied to test for differences in community composition among groups. Intergroup comparisons of bacterial abundance were performed using Kruskal–Wallis tests, as the data did not meet normality assumptions. The significance threshold was set at α = 0.05 for all analyses.

## 3. Results

### 3.1. Dietary p-Coumaric Acid Does Not Affect Worker Survival

**Figure 1:**
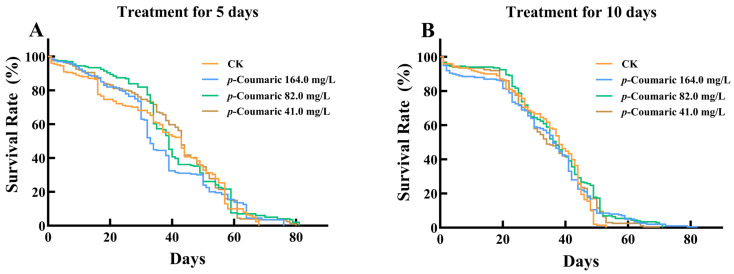
The survival curves of bee workers treated with *p*-Coumaric acid. (**A**) *p*-Coumaric acid treatment for 5 days. (**B**) *p*-Coumaric acid treatment for 10 days. CK, control group. *n* = 200 for each group.

Short-term dietary exposure to *p*-coumaric acid did not affect worker survival. Kaplan–Meier analysis indicated no significant differences in mortality between the CK and any of the *p*-coumaric acid treatment groups (164.0, 82.0, or 41.0 mg/L) after 5 days (Figure 1A; log-rank test: *χ*^2^ = 4.755; df = 3, 796; *p* = 0.19 > 0.05) or 10 days of exposure (Figure 1B; log-rank test: *χ*^2^ = 5.323; df = 3, 796; *p* = 0.15 > 0.05).

### 3.2. Effects of Dietary p-Coumaric Acid on Sucrose Solution Consumption

**Figure 2:**
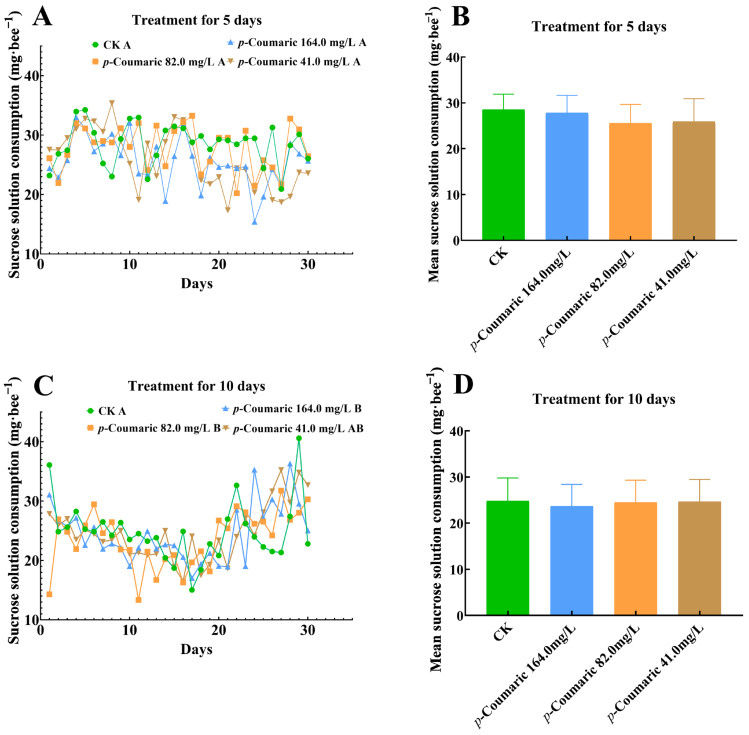
Sucrose solution consumption of *Apis cerana* workers. The line graph shows the sucrose solution consumption of honeybees under *p*-coumaric acid treatment for 5 days (**A**) and 10 days (**C**) over a 30-day period. The bar graph shows the mean sucrose solution consumption of honeybees under *p*-coumaric acid treatment for 5 days (**B**) and 10 days (**D**). Tested for differences between groups using two-way repeated measures ANOVA, with Bonferroni correction, α = 0.05; different capital letters following the group names indicate significant differences at the *p* < 0.01 level. CK, control group.

Analysis of dietary intake revealed that sucrose solution consumption was influenced by *p*-coumaric acid supplementation. In the 5-day exposure groups, sucrose consumption did not differ significantly among treatment groups at any concentration (Figure 2A; two-way repeated measures ANOVA: F = 0.70; df = 3, 16; *p* = 0.534 > 0.05). In comparison, in the 10-day exposure groups, sucrose solution consumption was significantly affected (Figure 2C; two-way repeated measures ANOVA: F = 4.51; df = 3, 16; *p* = 0.006 < 0.05). Bees receiving 164.0 mg/L and 82.0 mg/L *p*-coumaric acid consumed significantly less sucrose solution compared with the CK (both *p* < 0.01), whereas consumption in the 41.0 mg/L group did not differ from CK (*p* > 0.05). Despite these temporal changes, the mean sucrose intake per bee over the treatment period did not differ significantly among concentrations in either the 5-day group (Figure 2B; one-way ANOVA: F = 0.53; df = 3, 16; *p* = 0.67 > 0.05) or the 10-day group (Figure 2D; one-way ANOVA: F = 0.19; df = 3, 16; *p* = 0.90 > 0.05).

### 3.3. Effects of Dietary p-Coumaric Acid on the Gut Microbiota Abundance

**Figure 3:**
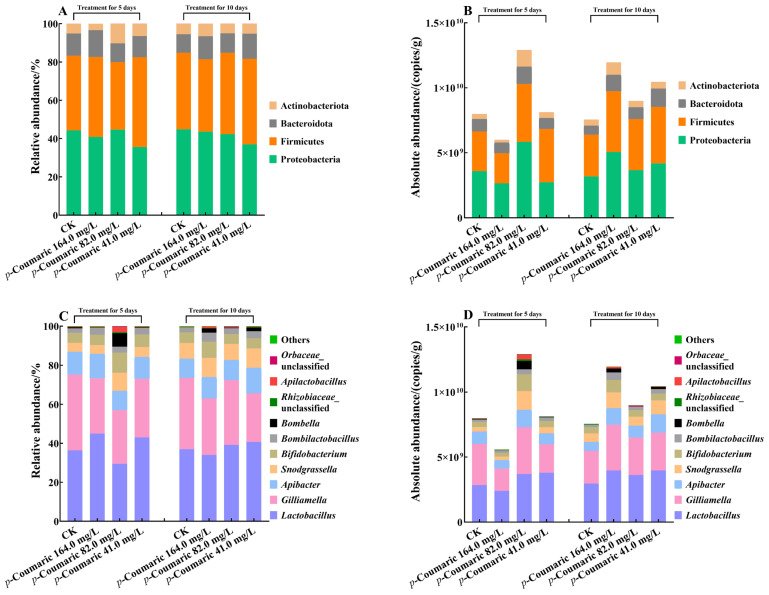
Taxonomic analysis of worker bee gut microbiota at phylum and genus levels. Relative abundance of dominant bacterial communities at the phylum level (**A**) and at the genus level (**C**); absolute abundance of dominant bacterial communities at the phylum level (**B**) and at the genus level (**D**). CK, control group.

The gut microbial community of worker bees was assessed at both phylum and genus levels. Relative abundance analysis indicated that gut microbiota composition was largely consistent across all experimental groups (Figure 3A,C). At the phylum level (Figure 3A), Proteobacteria (41.65%) and Firmicutes (40.98%) were the most abundant, followed by Bacteroidota (11.42%), Actinobacteriota (5.93%), and other rare phyla (0.02%) (Table S2). At the genus level (Figure 3C), the core bacterial taxa included *Lactobacillus* (38.09%), *Gilliamella* (31.04%), *Snodgrassella* (11.09%), *Apibacter* (7.45%), and *Bifidobacterium* (6.39%) (Table S3). Absolute abundance analysis showed that the total bacterial load in CK bees remained stable over time, with no significant difference observed between the 5-day (7.99 × 10^9^ ± 1.30 × 10^9^ copies/g) and 10-day exposure periods (7.57 × 10^9^ ± 2.74 × 10^9^ copies/g) (Kruskal–Wallis: H = 2.73; df = 1, 9; *p* = 0.60 > 0.05) (Figure 3B,D).

**Figure 4:**
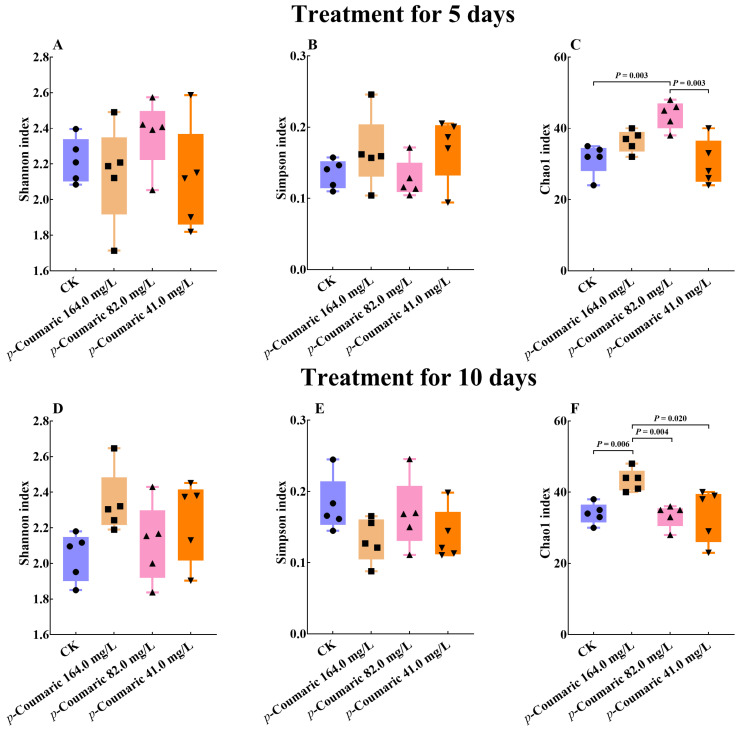
The Shannon, Simpson, and Chao1 indices across treatment groups. (**A**) Shannon index after 5 days exposure. (**B**) Simpson index after 5 days exposure. (**C**) Chao1 index after 5 days exposure. (**D**) Shannon index after 10 days exposure. (**E**) Simpson index after 10 days exposure. (**F**) Chao1 index after 10 days exposure. Group differences were tested by Kruskal–Wallis test, α = 0.05. CK, control group.

*p*-Coumaric acid exposure influenced gut microbial richness, as reflected by alpha diversity metrics. In the 5-day exposure groups, the Chao1 index showed significant differences among groups (Figure 4C; Kruskal–Wallis: H = 11.79; df = 3, 16; *p* = 0.01). The 82.0 mg/L group showed higher richness compared with the CK (*p* < 0.01) and 41.0 mg/L group (*p* < 0.01), while no significant difference was observed relative to the 164.0 mg/L group (*p* = 0.65 > 0.05). After 10 days of treatment (Figure 4F; Kruskal–Wallis: H = 10.79; df = 3, 16; *p* = 0.01), the 164.0 mg/L group showed significantly greater richness than all other groups (CK, *p* < 0.01; 82.0 mg/L, *p* < 0.01; 41.0 mg/L, *p* = 0.02 < 0.05). In comparison, neither Shannon (Kruskal–Wallis: 5 d: H = 2.76; df = 3, 16; *p* = 0.43 > 0.05; 10 d: H = 6.68; df = 3, 16; *p* = 0.08 > 0.05) nor Simpson (Kruskal–Wallis: 5 d: H = 3.90; df = 3, 16; *p* = 0.27 > 0.05; 10 d: H = 5.29; df = 3, 16; *p* = 0.15 > 0.05) diversity indices were not significantly affected by treatment at either timepoint (Figure 4A,B,D,E).

**Figure 5:**
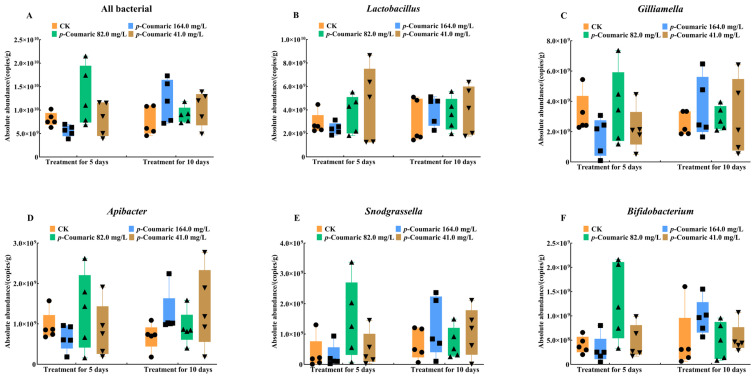
The absolute abundance of (**A**) total bacterial copies and the five core bacterial genera, (**B**) *Lactobacillus*, (**C**) *Gilliamella*, (**D**) *Apibacter*, (**E**) *Snodgrassella* and (**F**) *Bifidobacterium* of *A. cerana* workers. Differences among groups were examined using Kruskal–Wallis test, α = 0.05. CK, control group.

Total gut bacterial load and the absolute abundances of genera, including *Lactobacillus*, *Gilliamella*, *Snodgrassella*, *Apibacter*, and *Bifidobacterium*, were quantified (Figure 5). The results showed that *p*-coumaric acid treatment had no significant effect on total bacterial copy numbers (Figure 5A; Kruskal–Wallis: 5d H = 6.95; df = 3, 16; *p* = 0.07 > 0.05; 10d H = 4.01; df = 3, 16; *p* = 0.26 > 0.05) and insignificant differences were observed in the absolute abundances of *Lactobacillus* (Figure 5B; Kruskal–Wallis: 5d H = 1.10; df = 3, 16; *p* = 0.78 > 0.05; 10d H = 1.77; df = 3, 16; *p* = 0.62 > 0.05), *Gilliamella* (Figure 5C; Kruskal–Wallis: 5d H = 3.62; df = 3, 16; *p* = 0.31 > 0.05; 10d H = 1.34; df = 3, 16; *p* = 0.72 > 0.05), *Apibacter* (Figure 5D; Kruskal–Wallis: 5d H = 1.54; df = 3, 16; *p* = 0.67 > 0.05; 10d H = 5.65; df = 3, 16; *p* = 0.13 > 0.05), *Snodgrassella* (Figure 5E; Kruskal–Wallis: 5d H = 3.80; df = 3, 16; *p* = 0.28 > 0.05; 10d H = 1.51; df = 3, 16; *p* = 0.68 > 0.05), and *Bifidobacterium* (Figure 5F; Kruskal–Wallis: 5d H = 3.77; df = 3, 16; *p* = 0.29 > 0.05; 10d H = 4.99; df = 3, 16; *p* = 0.17 > 0.05) in *A. cerana*.

**Figure 6:**
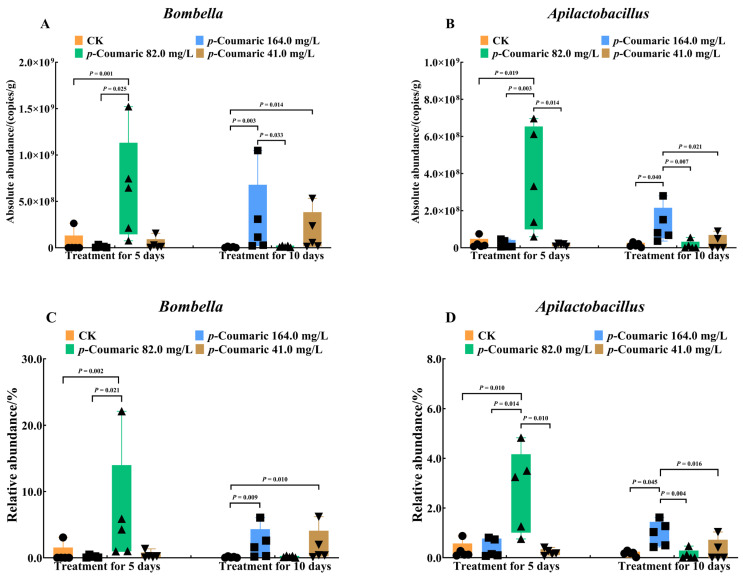
Quantification of absolute and relative abundance for differential non-dominant bacterial genera in *A. cerana* workers after *p*-coumaric acid exposure. (**A**) Absolute abundance of *Bombella*. (**B**) Absolute abundance of *Apilactobacillus*. (**C**) Relative abundance of *Bombella*. (**D**) Relative abundance of *Apilactobacillus*. Differences among groups were examined using Kruskal–Wallis test, α = 0.05. CK, control group.

In comparison, non-core bacterial taxa displayed distinct responses that varied with *p*-coumaric acid concentration and exposure duration, primarily reflected by changes in the absolute and relative abundances of *Bombella* and *Apilactobacillus* (Figure 6). After 5 days of exposure, both absolute and relative abundances of *Bombella* differed significantly among groups (Figure 6A,C; absolute: H = 10.88; df = 3, 16; *p* = 0.01; relative: H = 10.69; df = 3, 16; *p* = 0.01). The 82.0 mg/L group exhibited higher *Bombella* levels than CK (both absolute and relative *p* < 0.01) and the 164.0 mg/L group (absolute: *p* = 0.025 < 0.05; relative: *p* = 0.021 < 0.05). In the 10-day treatment, *Bombella* abundance increased significantly in the 164.0 mg/L group compared to the CK and the 82.0 mg/L group (Figure 6A,C; absolute: H = 11.58, df = 3, 16, *p* = 0.01; 164.0 mg/L vs. CK, *p* < 0.01; vs. 82.0 mg/L, *p* = 0.03; relative: H = 10.57, df = 3, 16, *p* = 0.01; 164.0 mg/L vs. CK, *p* < 0.01; vs. 82.0 mg/L, *p* = 0.01), whereas CK remained lower than the 41.0 mg/L group for both absolute and relative abundances (both *p* = 0.01). For *Apilactobacillus*, significant differences were detected after 5 days of exposure (Figure 6B,D; absolute: H = 10.62; df = 3, 16; *p* = 0.01; relative: H = 9.62; df = 3, 16; *p* = 0.02 < 0.05). The 82.0 mg/L group exhibited higher absolute and relative abundances than CK (absolute: *p* = 0.02 < 0.05; relative: *p* = 0.01), the 164.0 mg/L group (absolute: *p* < 0.01; relative: *p* = 0.01), and the 41.0 mg/L group (absolute and relative *p* = 0.01). After 10 days, *Apilactobacillus* abundance was highest in the 164.0 mg/L group (Figure 6B,D; absolute: H = 8.66, df = 3, 16, *p* = 0.03; vs. CK, *p* = 0.04; vs. 82.0 mg/L, *p* < 0.01; vs. 41.0 mg/L, *p* = 0.02 < 0.05; relative: H = 9.49, df = 3, 16, *p* = 0.02 < 0.05; vs. CK, *p* = 0.045; vs. 82.0 mg/L, *p* < 0.01; vs. 41.0 mg/L, *p* = 0.02 < 0.05).

**Figure 7:**
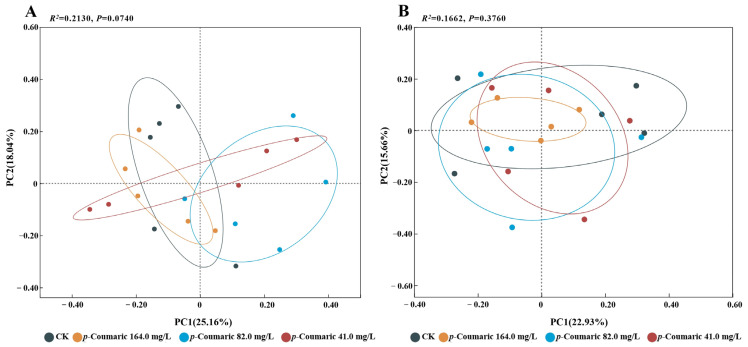
Principal coordinate analysis (PCoA) was performed after 5 (**A**) and 10 (**B**) days of exposure. Group differences were tested by PERMANOVA with α = 0.05. CK, control group.

PCoA indicated that no significant differences were observed after either 5 days (R^2^ = 0.21, F = 1.44, *p* = 0.07) or 10 days of *p*-coumaric acid exposure (R^2^ = 0.17, F = 1.06, *p* = 0.38 > 0.05) (Figure 7). These results reveal that *p*-coumaric acid supplementation minimal impact influence on overall gut microbiota structure.

## 4. Discussion

Dietary phytochemicals play essential roles in honeybee physiology, contributing to increased longevity and improved tolerance to pesticides [33,52]. Elucidating the mechanisms by which these compounds influence host physiological functions through interactions with the gut microbiota is therefore crucial for understanding their role in maintaining honeybee health. This study evaluated the effects of dietary *p*-coumaric acid on *A. cerana* survival, sucrose solution consumption, and gut microbial composition using absolute quantification sequencing. The results demonstrated that short-term exposure to *p*-coumaric acid did not affect worker survival; but caused temporal changes in sucrose solution consumption and selectively affected non-core bacterial taxa, while the overall composition of the core gut microbiota remained unchanged.

Compared with previous observations in which a 5-day dietary supplementation with 82.0 mg/L *p*-coumaric acid significantly extended the lifespan of *A. cerana* workers [46], the current study detected no such survival benefit. This discrepancy is likely attributable to differences in the bees’ physiological status across studies. In the earlier investigation, newly emerged workers received phytochemical treatment immediately, before the establishment of a fully colonized gut microbiota. In this study, workers were allowed to establish a stable natural gut microbiome for 7 days in the hive before *p*-coumaric acid exposure. The absence of a longevity effect in colonized bees indicates that the physiological outcomes of dietary phytochemicals are not solely inherent to the compounds but are strongly modulated by interactions with the gut microbiota. This highlights the gut microbiome as a key modulator of the host’s response to dietary phytochemicals [29]. These results emphasize the importance of considering gut microbial colonization status in future studies assessing the effects of dietary phytochemicals on bees.

The lack of significant survival effects across all tested concentrations (41.0–164.0 mg/L) indicates that *A. cerana* tolerates *p*-coumaric acid well over a 10-day exposure period. This is consistent with previous findings on other phytochemicals, such as quercetin, which also showed no acute toxicity at naturally occurring dietary levels [33,46]. Sucrose consumption showed a temporal shift, with no effect at 5 days but significant changes at 10 days, suggesting that *p*-coumaric acid may elicit gradual physiological adaptations. This delayed response could result from cumulative effects on metabolic pathways or gustatory perception. Such adaptations, as reflected in altered sucrose intake, in turn, may influence energy-dependent behaviors, including foraging motivation [37,53,54], highlighting the need to investigate the broader behavioral consequences of prolonged phytochemical exposure.

Exposure to *p*-coumaric acid was selectively modulated in non-core gut bacteria, specifically *Bombella* and *Apilactobacillus*, whereas the abundance of core genera (*Lactobacillus*, *Gilliamella*, *Snodgrassella*, *Apibacter*, and *Bifidobacterium*) remained stable in *A. cerana*. This pattern contrasts with observations in *A. mellifera*, where dietary *p*-coumaric acid significantly increased the abundance of core genera, including *Snodgrassella* and *Lactobacillus* [30]. The discrepancy supports the hypothesis that dietary *p*-coumaric acid drives species-specific modulation of the gut microbiota, likely reflecting inherent differences in microbial architecture between the two honeybee species. For example, *A. cerana* typically harbors a higher *Apibacter* abundance, whereas *A. mellifera* is predominantly colonized by *Commensalibacter* [18]. The differential responses, non-core bacteria shifting in *A. cerana* and core bacteria in *A. mellifera*, indicate that the same phytochemical may target distinct microbial compartments depending on the host species. This further suggests that *A. cerana* and *A. mellifera* have evolved distinct microbial strategies for processing phytochemicals, which may influence their tolerance to dietary phytochemicals. These findings underscore the necessity of accounting for pollinator species and their specific gut microbial ecosystems when evaluating the ecological and physiological effects of phytochemicals.

The persistence of core microbiota stability and consistent diversity metrics indicates a strong resistance of the gut community to perturbation by *p*-coumaric acid. This differs from previous observations for quercetin, another dietary phytochemical in bees, which at high concentrations (151.2 and 75.6 mg/L) transiently suppressed total bacterial load and *Lactobacillus* abundance after 5 days of exposure. Microbial recovery was observed by day 9, accompanied by an increase in *Gilliamella* at a lower concentration (37.8 mg/L) [45]. The recovery, particularly the enrichment of *Gilliamella*, suggests a functional role for this symbiont in quercetin metabolism, consistent with its known ability to process diverse dietary compounds and sugars [23,55,56]. Dietary quercetin has also been shown to upregulate host detoxification genes, such as cytochrome P450 monooxygenases [57,58], a process that can be further modulated by the gut microbiota [57,59]. These complementary mechanisms, microbial metabolism and host enzymatic detoxification, act together to maintain gut homeostasis under xenobiotic challenge. In the current study, *p*-coumaric acid selectively modulated non-core bacteria, including *Bombella* and *Apilactobacillus*, without perturbing core genera. This differential response may reflect distinct detoxification pathways or differences in phytochemical bioavailability. As a relatively simple phenolic acid, *p*-coumaric acid may be readily metabolized by microbial enzymes, such as phenolic acid decarboxylases, limiting its impact on the core microbiome [23,58]. *Bombella* is involved in nitrogen metabolism and larval nutrition, supporting protein utilization and brood development [56], while *Apilactobacillus*, contributes to carbohydrate fermentation and stress tolerance [16]. Their increased abundance in response to *p*-coumaric acid suggests a potential role in phytochemical metabolism and maintenance of gut homeostasis under dietary stress.

The overall gut microbiota structure remained largely stable following *p*-coumaric acid exposure, although specific non-core genera displayed changes. The Chao1 index was significantly increased in the 82.0 mg/L group after 5 days and in the 164.0 mg/L group after 10 days, indicating that *p*-coumaric acid supplementation at particular concentrations and durations facilitates the colonization or detection of additional bacterial taxa in the honeybee gut. However, no significant differences were observed in the Shannon and Simpson indices across all groups and timepoints, suggesting that *p*-coumaric acid did not disrupt community evenness. Beta diversity analyses further confirmed that the overall gut microbial community structure did not diverge significantly between control and *p*-coumaric acid-treated bees after either 5- or 10-day exposure. This stability reflects the gut microbial community’s resilience, which is likely essential for sustaining host health under fluctuating dietary conditions. These findings are consistent with previous studies indicating that the honeybee gut microbiota can rapidly adapt to dietary phytochemicals through enzymatic diversification and horizontal gene transfer [56]. The results further suggest that non-core bacteria serve as a flexible metabolic reservoir, buffering the host against dietary perturbations, a key adaptation for social bees that depend on nutritionally variable floral resources.

Although this study demonstrates that *p*-coumaric acid influences sucrose solution consumption and selectively modulates non-core gut bacteria without altering the core microbiota, some limitations should be acknowledged. The short experimental duration may not reflect long-term effects on colony performance, and cage-rearing conditions do not replicate natural hive behaviors, such as foraging and social interactions, which influence gut microbial dynamics. Furthermore, the lack of functional omics and targeted molecular data limits the mechanistic insights into *p*-coumaric acid metabolism. Future studies should aim to elucidate the regulatory effects of *p*-coumaric acid on *Bombella* and *Apilactobacillus* using targeted molecular techniques and multi-omics approaches. They should also extend exposure periods and incorporate field-based experimental designs to better define functional interactions between dietary phytochemicals and the honeybee gut microbiome.

## 5. Conclusions

In conclusion, short-term dietary supplementation with *p*-coumaric acid does not impact the survival of *A. cerana* workers or the composition of the core gut microbiota. *p*-Coumaric acid exposure, however, selectively alters specific non-core bacterial taxa, and influences sucrose solution consumption in the 10-day exposure groups. These results indicate that *p*-coumaric acid exerts context-dependent effects on physiology and gut microbial composition without compromising host viability or core microbiome stability in *A. cerana*.

## Data Availability

The original contributions presented in the study are included in the article/Supplementary Material. Further inquiries can be directed to the corresponding author.

## Supplementary Materials

The following supporting information can be downloaded at: https://www.mdpi.com/article/10.3390/insects17040371/s1, Table S1: The standard curve based on read counts versus spike-in DNA copy number of each sample. Table S2: Relative abundance of major bacterial phyla based on copy number analysis; Table S3: Relative abundance of major bacterial genera based on copy number analysis; Figure S1: The relative abundance of dominant bacterial genera of worker bees.
