# Supplementary material for: Dietary p-Coumaric Acid Modulates Non-Core Gut Microbiota and Sucrose Solution Consumption in Apis cerana
Source: Insects. 2026 Apr 1;17(4):371. doi: 10.3390/insects17040371 (PMC13115751; doi:10.3390/insects17040371)
Supplement: Supplementary file 1 [file insects-17-00371-s001.zip › insects-4180434-supplementary.pdf]

Table S1. The standard curve based on read counts versus spike-in DNA copy number of each sample

Value	5d CK	5d PC1	5d PC2	5d PC3	9d CK	9d PC1	9d PC2	9d PC3
**a**	0.9912	0.9983	0.9758	0.9990	0.9974	0.9968	0.9783	1.0004
**b**	1.9418	1.8294	2.1350	1.9342	2.1295	2.2952	2.2123	2.0552
R^2	0.9889	0.9877	0.9824	0.9894	0.9866	0.9874	0.9858	0.9843

a, the slope of the standard curve; b, the intercept of the standard curve; R^2, the determination coefficient of the standard curve. CK, control group; PC1, 164.0 mg/L *p*-coumaric acid treatment group; PC2, 82.0 mg/L *p*-coumaric acid treatment group; PC3, 41.0 mg/L *p*-coumaric acid treatment group.

Table S2. Relative abundance of major bacterial phyla based on copy number analysis

Taxon	Proteobacteria	Firmicutes	Bacteroidota	Actinobacteriota	Others
**5d CK**	44.2835%±4.3338%	39.0128%±5.1824%	11.6097%±1.2160%	5.0792%±1.0613%	0.0148%±0.0092%
**5d PC1**	40.8934%±9.3240%	41.7279%±6.5395%	13.9996%±3.5734%	3.3346%±2.2681%	0.0444%±0.0179%
**5d PC2**	44.6598%±4.3282%	35.2865%±3.8972%	9.8107%±2.7120%	10.2252%±2.6352%	0.0179%±0.0064%
**5d PC3**	35.6460%±6.3655%	46.8529%±6.2802%	11.0654%±3.4936%	6.3972%±2.0260%	0.0386%±0.0146%
**10d CK**	44.8199%±4.7328%	39.9894%±4.8546%	9.6867%±2.5905%	5.4959%±2.4501%	0.0082%±0.0082%
**10d PC1**	43.5856%±3.1269%	37.8400%±2.9562%	12.0132%±1.4553%	6.5381%±0.7716%	0.0231%±0.0112%
**10d PC2**	42.3167%±5.9389%	42.4692%±4.4409%	10.1490%±1.5741%	5.0626%±1.5741%	0.0024%±0.0024%
**10d PC3**	37.0004%±8.2549	44.6642%±10.5749%	13.0471%±1.0854%	5.2844%±1.0854%	0.0039%±0.0039%
**Average**	40.3678%	42.1266%	11.0950%	6.3910%	0.0196%

CK, control group; PC1, 164.0 mg/L *p*-coumaric acid treatment group; PC2, 82.0 mg/L *p*-coumaric acid treatment group; PC3, 41.0 mg/L *p*-coumaric acid treatment group.

Table S3. Relative abundance of major bacterial genera based on copy number analysis

Taxon	*Lactobacillus*	*Gilliamella*	*Snodgrassella*	*Apibacter*	*Bifidobacterium*	*Orbaceae*_unclassified	*Bombella*	*Rhizobiaceae*_unclassified	*Bombilactobacillus*	*Apilactobacillus*	Others
**5d CK**	36.3581%±5.0605%	38.8471%±4.4807%	4.6017%±3.2412%	11.5947%±1.2212%	5.0792%±1.0613%	0.0635%±0.0635%	0.6161%±0.6118%	-	2.3617%±0.7430%	0.2930%±0.1483%	0.1850%±0.0579%
**5d PC1**	44.9907%±5.9631%	28.3250%±8.9392%	4.5046%±2.3484%	12.4485%±3.5738%	5.2901%±2.2670%	0.0593%±0.0593%	0.1840%±0.0814%	-	3.5923%±0.7414%	0.3687%±0.1640%	0.2368%±0.0446%
**5d PC2**	29.4875%±3.1883%	27.4923%±5.5607%	9.3558%±2.5004%	9.8107%±2.7120%	10.2252%±2.6352%	0.2914%±0.2324%	6.8372%±3.9334%	0.5994%±0.5946%	3.0634%±0.6849%	2.7156%±0.7526%	0.1216%±0.0223%
**5d PC3**	43.0254%±6.3968%	30.0145%±7.6200%	5.1449%±2.1224%	11.0654%±3.4936%	6.3972%±2.0260%	0.0028%±0.0028%	0.3907%±0.2429%	-	3.5268%±0.7173%	0.2113%±0.0590%	0.2211%±0.0855%
**10d CK**	36.9857%±4.3404%	36.5449%±6.1823%	8.1047%±1.8334%	9.6762%±2.5905%	5.4920%±2.4496%	-	0.0848%±0.0425%	-	2.5248%±0.4219%	0.1692%±0.0430%	0.4176%±0.2835%
**10d PC1**	34.0138%±2.3750%	28.9876%±3.9725%	9.7361%±3.0655%	10.9378%±1.4561%	8.2923%±0.7716%	0.0146%±0.0103%	2.1315%±1.0777%	-	4.7168%±0.7478%	0.9678%±0.2265%	0.2018%±0.0366%
**10d PC2**	39.1688%±5.7923%	33.2100%±5.6549%	8.2894%±3.0629%	10.1445%±2.1779%	5.0626%±1.5741%	0.5963%±0.5963%	0.1529%±0.0549%	-	2.9770%±0.6289%	0.1149%±0.0896%	0.2836%±0.5963%
**10d PC3**	40.7198%±9.8577%	24.8996%±7.0138%	9.8307%±2.5855%	13.0444%±3.2482%	5.2838%±1.0849%	-	1.8019%±1.1455%	0.3878%±0.3878%	3.6084%±0.7314%	0.2881%±0.2024%	0.1355%±0.0333%
**Average**	38.2520%	30.2537%	7.3519%	11.2923%	6.5186%	0.1468%	1.7306%	0.1410%	3.4066%	0.7085%	0.1979 %

- means no copies of this bacterial genus were measured in the gut sample. CK, control group; PC1, 164.0 mg/L *p*-coumaric acid treatment group; PC2, 82.0 mg/L *p*-coumaric acid treatment group; PC3, 41.0 mg/L *p*-coumaric acid treatment group.

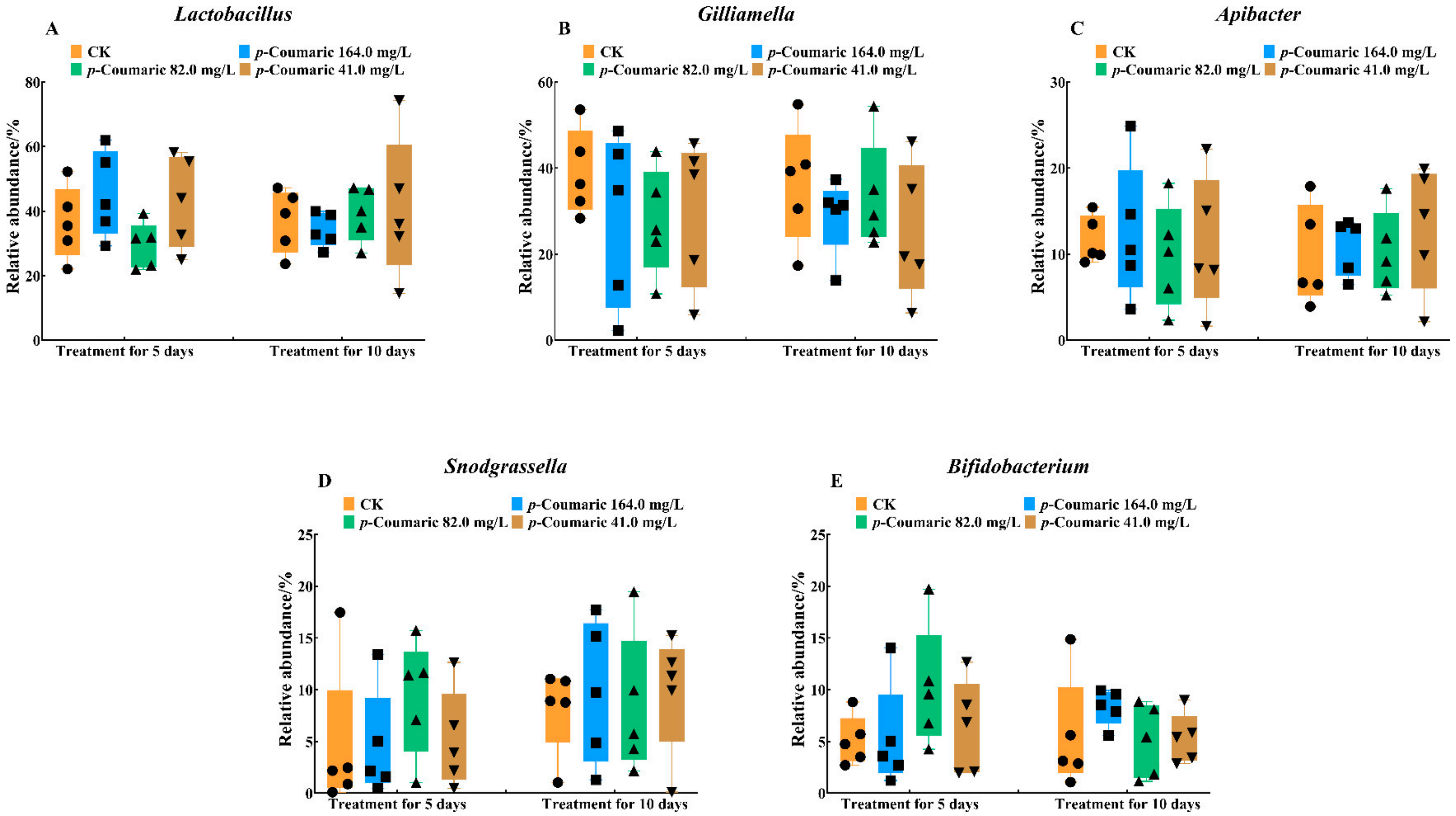

Figure S1. The relative abundance of dominant bacterial genera of worker bees.

The relative abundance of the five most abundant bacterial genera of *A. cerana* workers were measured as copies of the 16S rRNA gene. (**A**) *Lactobacillus*; (**B**) *Gilliamella*; (**C**) *Apibacter*; (**D**) *Snodgrassella*; (**E**) *Bifidobacterium*. Tested for differences between groups using Kruskal–Wallis analysis, $\alpha = 0.05$. CK, control group.
